# Changes in Nutritional Status during Induction Phase and Their Association with Fever and Minimal Residual Disease in Paediatric Acute Lymphoblastic Leukaemia

**DOI:** 10.3390/medicina59061008

**Published:** 2023-05-24

**Authors:** Sigita Gustaitė, Veronika Everatt, Ignė Kairienė, Ramunė Vaišnorė, Jelena Rascon, Goda Elizabeta Vaitkevičienė

**Affiliations:** 1 Faculty of Medicine, Vilnius University, 03101 Vilnius, Lithuania; 2 Reference Centre for Oncohaematological Diseases at the Haematology, Oncology and Transfusion Medicine Centre, Vilnius University Hospital Santaros Klinikos, 08661 Vilnius, Lithuania

**Keywords:** acute lymphoblastic leukaemia, nutrition, body mass index, fever, minimal residual disease

## Abstract

*Background and objectives:* Acute lymphoblastic leukaemia (ALL) is associated with a cytokine imbalance and oxidative stress, which can be aggravated by malnutrition. Malnutrition, defined by the World Health Organisation (WHO) as obesity or undernutrition, can affect treatment complications and outcomes. Therefore, we aimed to analyse the change in the body mass index (BMI) *z*-score during induction, as well as evaluate the impact of childhood malnutrition on fevers at an ALL presentation and early response to therapy. *Methods*: An observational cohort study of 50 consecutive children with ALL, diagnosed in 2019–2022, was performed. Patients were divided into age groups of 0–5, 6–11, and 12–17 years. BMI-for-age *z*-scores were used to define undernutrition and overnutrition according to WHO growth standards. *Results:* The number of patients with an abnormal BMI increased from 3 (6%) at diagnosis to 10 (20%) at the end of induction (from 2 (4%) to 6 (12%) in overweight/obese, and from 1 (2%) to 4 (8%) in underweight patients). At the end of induction, all overweight/obese patients were 0–5 years old. On the other hand, a statistically significant decrease in the mean BMI *z*-score among patients aged 12–17 was observed (*p* = 0.005). The mean BMI *z*-score differed statistically significantly among children aged 0–5 presenting with and without fever (*p* = 0.001). The minimal residual disease (MRD) level at the end of induction was not related to BMI at diagnosis. *Conclusions*: Despite the use of steroids, adolescents are prone to losing weight during an ALL induction, in contrast to preschool children, who tend to gain weight under the same treatment. BMI at diagnosis was related to a fever of ≥38 °C (at ALL presentation) in the 0–5 age group. The results emphasise the importance of careful nutritional status monitoring, with younger and older children as important target groups for weight gain and weight loss interventions, respectively.

## 1. Introduction

Acute lymphoblastic leukaemia (ALL) is the most common paediatric malignancy, comprising of 25% of cancers in children younger than 15 years of age. Survival rates for children treated according to contemporary chemotherapy protocols approach 90% [1]. However, the treatment may cause serious complications, and the incidence of treatment-related mortality in ALL is reported to be between 2% and 4%. Around 1% of treatment-related mortality occurs during induction [2,3,4,5,6].

Fever is a common manifestation of paediatric ALL and can be caused by both leukaemia-related pyrogenic reactions and infection. ALL is associated with disruptions in a cytokine balance, including elevation in the levels of tumour necrosis factor alpha (TNF-α) and interleukin 1 (IL-1) [7]. Infection is another possible cause of fever at presentation in paediatric ALL patients. The risk of infection is increased by malnutrition, which weakens the immune response through changes in cytokine function, micronutrient deficiencies, damaged functions of phagocytes, and complement system disorders [8,9,10,11]. Moreover, malnutrition and obesity, specifically linked to chronic low-grade systemic inflammation, are associated with alterations in cytokine levels and acute phase reactants, and they can likewise contribute to the risk of fever [12].

It has been suggested that a worse early response to therapy in childhood ALL, defined as persistent minimal residual disease (MRD) at the end of induction, might be linked to higher levels of oxidative markers [13]. Oxidative stress and the production of free radicals are closely related to an enhanced cytokine production and cytokine imbalance [14]. Both oxidative stress and cytokine imbalance are aggravated by a poor nutritional status, as both wasting and obesity can cause an elevation in free-radical levels, as well as cytokine balance disruptions [15,16,17]. During ALL induction, patients undergo significant changes in nutritional status due to multiple reasons. The use of glucocorticoids in the backbone of induction is a well-known factor leading to several adverse metabolic effects, such as weight gain, altered fat distribution, dyslipidaemia, insulin resistance, and hyperglycaemia, all of which can be aggravated by enhanced energy intake and reduced physical activity during treatment [18,19,20].

Our study aimed to analyse the change in body mass index (BMI) during induction, as well as the impact of BMI on fever at diagnosis and early response to chemotherapy. We hypothesised that children with ALL would tend to gain weight during the first weeks of treatment. We focused on the induction period only—a relatively short but challenging treatment phase.

## 2. Materials and Methods

### 2.1. Study Population and Design

A population-based observational cohort study was performed. All consecutive patients diagnosed with childhood ALL from 2019 to 2022 at the Centre for Paediatric Oncology and Haematology at Vilnius University Hospital Santaros Klinikos were enrolled. The diagnostics and treatment of paediatric ALL in Lithuania have been centralised at our institution since the early 1980s. Patients’ baseline characteristics, as well as clinical, laboratory, and treatment data from the induction period, were retrospectively retrieved from medical records for the 2019–2021 period or prospectively collected for the 2021–2022 period.

The study cohort was divided into 3 age categories: 0–5 (preschool), 6–11 (prepubertal), and 12–17 years (pubertal). A cut-off of 12 years was selected to discern the oldest age group, as 12 years is the average age of pubertal onset in Lithuania [21].

Weight and height were evaluated at diagnosis and at 4 timepoints during the induction phase—on days 8, 15, 22, and 29 of the protocol. The ALL induction treatment is described below in detail. The World Health Organisation (WHO) defines malnutrition as insufficient or excessive nutrient intake, as well as an imbalance of essential nutrients or impaired nutrient utilisation [22]. Assessment of nutritional status was based on the BMI *z*-score, which was calculated using the WHO Anthro (version: 3.2.2, Geneva, Switzerland) and AnthroPlus (version: 1.0.4, Geneva, Switzerland) software. The BMI-for-age *z*-score was determined according to the WHO growth standards, and cut-off points of between −2 and 2, less than −2, and more than 2 were used to define normal weight, underweight, and overweight or obese patients, respectively.

Data on confirmed infections, established by a positive infection site culture, viral antigen test, or molecular test, were collected if available. Fever at diagnosis was defined as fever confirmed at the time of hospitalization and registered in medical files before the beginning of the induction treatment. Patients were stratified into two groups according to febrile temperature (<38 °C and ≥38 °C). The axillary temperature was measured by experienced medical personnel using certified digital thermometers twice a day (in the morning and evening), or more frequently whenever a temperature of ≥37.5 °C was recorded or fever was clinically suspected.

Data on the MRD at the end of induction (day 29) were retrieved, with MRD negativity defined as undetectable blast cells in the bone marrow by both flow cytometry and polymerase chain reaction, or only by the former when the latter was not available. The day 29 MRD for one female patient was not evaluated as she died during induction prior to day 29 because of an invasive fungal pulmonary infection. However, she continued dexamethasone until day 28; therefore, her data were included.

Written consent from a legal guardian was obtained for the patients, and ethical approval was obtained from the Vilnius Regional Committee of Biomedical Research (approval no. 2022/5-1436-908).

### 2.2. Statistical Analysis

The following descriptive statistics were calculated: the median and interquartile range (IQR) for age, the mean and standard deviation (SD) for other continuous variables, and the frequency with percentages for categorical variables.

The association between different categorical variables was tested using chi-square and Fisher’s exact tests. To evaluate the difference in the mean BMI *z*-score between the age groups, one-way analysis of variance (ANOVA) was used. The paired *t*-test was used to determine the change in the mean BMI *z*-score from diagnosis to the end of induction treatment. To evaluate the differences between the mean or median BMI *z*-scores at diagnosis (continuous variable) in patients categorised according to body temperature at presentation and different MRD status on day 29, independent-sample *t*-tests or two-sample Wilcoxon tests were used on the basis of the result of the Shapiro–Wilk normality test. A *p*-value of <0.05 was considered to be statistically significant. Statistical analysis was performed using R Commander (version: 4.1.1, Ontario, Canada).

## 3. Treatment

Patients received treatment according to the ALLTogether Pilot chemotherapy protocol. Children were stratified into 2 risk groups at induction: (i) National Cancer Institute (NCI) standard-risk patients (B-ALL, age at diagnosis <10 years, and leukocyte count <50 × 10^9^/L) received dexamethasone (6 mg/m^2^ on days 1–28, tapered), vincristine (1.5 mg/m^2^, maximum single dose 2 mg on days 1, 8, 15, and 22), PEG-asparaginase (1500 IU/m^2^ dose on days 4 and 18), and intrathecal methotrexate adjusted to age; (ii) NCI high-risk patients (all T-ALL, B-ALL if ≥10 years, and/or leukocyte count ≥50 × 10^9^/L) additionally received daunorubicin (25 mg/m^2^ dose on days 1, 8, 15, and 22).

A five-month-old infant was treated according to the Interfant-21 protocol. The patient received a daily 6 mg/m^2^ dexamethasone dose for 28 days (and tapered), as well as weekly vincristine, two doses of daunorubicin, daily cytarabine for 14 days, two doses of PEG-asparaginase every 14 days and intrathecal methotrexate, cytarabine and methylprednisolone to induce remission.

## 4. Results

A total of 50 consecutive patients (28 males and 22 females; median age 5.8; range 0.4–15.8 years, IQR = 6.1) were included in the analysis. No statistically significant differences were observed in terms of gender, immunophenotype, cytogenetics, or leukocyte count group among different age groups at diagnosis (Table 1).

### 4.1. BMI Change during Induction

**Figure 1:**
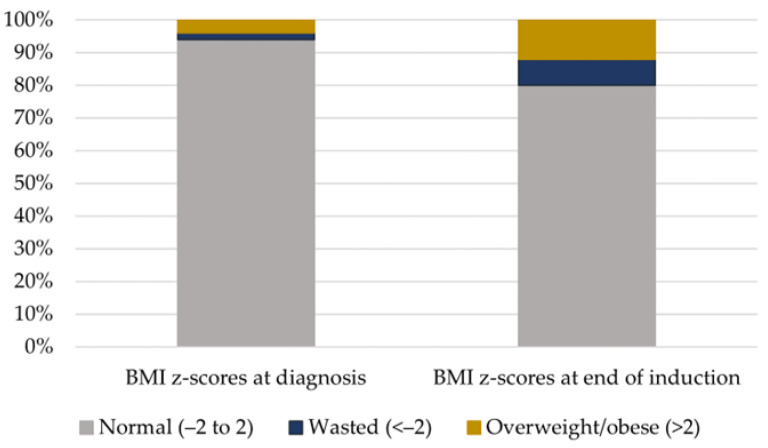
Frequency of abnormal BMI *z*-scores before and after induction.

At the time of diagnosis, 3 patients (6%) presented with an abnormal BMI *z*-score; 1 (2%) was underweight, and 2 (4%) were overweight/obese. At the end of induction (day 29 of the protocol), the number of patients with an abnormal BMI *z*-score more than tripled to 10 (20%); 2 boys and 4 girls were overweight/obese, while 2 boys and 2 girls were underweight (Figure 1). An abnormal BMI *z*-score at the end of induction was more prevalent among small children aged 0–5 years; among 8 (30.8%) patients with an abnormal BMI *z*-score on day 29, 2 (7.7%) were underweight, and 6 (23.1%) were overweight/obese. Only 2 children (8.3%) aged >5 years had an abnormal BMI *z*-score on day 29 (Table 1).

**Figure 2:**
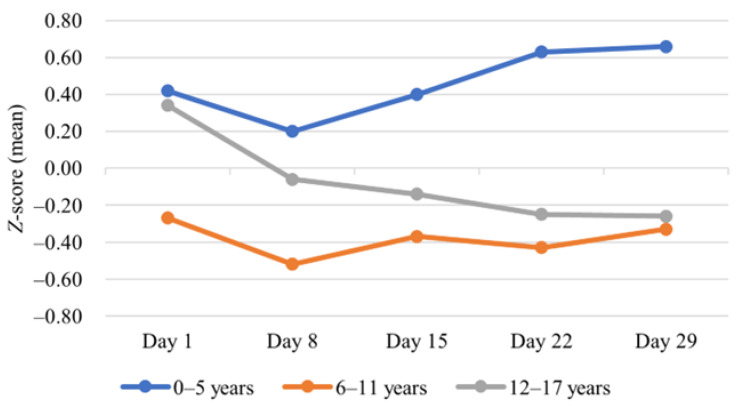
BMI change during induction treatment.

An increase in BMI *z*-score was most prominent among the youngest children (0–5 age group), with a mean BMI-for-age *z*-score of 0.4 at diagnosis and 0.7 at the end of induction, but the change was not statistically significant. In contrast, a statistically significant decrease (0.3 to −0.3, *p* = 0.005) was observed in BMI-for-age *z*-scores among adolescents (12–17 age group) (Figure 2, Table 1).

### 4.2. BMI and Fever

Nineteen patients presented with fever (≥38 °C) at diagnosis; among them, only one had an infection, confirmed by laboratory tests (Table 1). In the 0–5 age group, a statistically significant difference was detected in the average BMI *z*-score between children with a temperature of <38 °C vs. ≥38 °C at diagnosis (*p* = 0.001), which was not observed in other age groups (Table 2).

### 4.3. BMI and MRD

At the end of induction, 19 (38.8%) patients had nondetectable MRD, while 30 (61.2%) were MRD-positive (Table 1). There was no statistically significant difference in average BMI *z*-score among children with a different MRD status on day 29 (Table 2).

## 5. Discussion

Undernutrition is a widespread childhood problem in low-income countries; however, the scale of malnutrition should not be underestimated in high-income regions. The proportion of malnourished children aged over 2 years in Lithuania in 2021 was 36.3%, with 21.7% overweight or obese and 14.6% underweight [23]. Malnutrition in ALL patients has been associated with reduced survival, increased drug toxicity, and treatment-induced complications [24]. However, research results are conflicting, and a clear association between the patient’s nutritional status and ALL outcomes needs to be established [25,26].

Malnutrition in children with ALL may be the result of an imbalanced diet and lifestyle, a consequence of cancer and/or its treatment, or both [27]. In our study, the number of patients with an abnormal BMI *z*-score increased from the start of treatment to the end of induction. Importantly, an abnormally high BMI *z*-score at the end of induction was more prevalent among small children aged 0–5 years in our study compared to older patients. Similarly to our research, other studies revealed that younger children, particularly those with a healthy or lower BMI at diagnosis, were most likely to gain weight during treatment [28,29]. Another study performed a univariate logistic regression analysis that revealed younger age to be a risk factor for an increase in BMI standard deviation after induction chemotherapy [30].

The different BMI change patterns among the three age groups observed in our study during induction could be attributed to several factors. The energy metabolism of the whole body is closely related to the amount of water, fat, muscle, and bone tissue. The body composition changes constantly during infancy, childhood, and adolescence, playing an important role in age-related metabolism differences [31]. For example, water and fat account for a larger proportion of body mass in infants compared to older children (although the latter increases in females during puberty). However, the decrease in total body water with age is caused entirely by a decline in the percentage of extracellular water, since the percentage of intracellular water increases [32]. Glucocorticoids have a mineralocorticoid effect, which causes salt and water retention, with water accumulation occurring in the extracellular space [33,34]. This mechanism might explain the more prominent increase in BMI among younger children observed in our study, although dexamethasone has only a minimal mineralocorticoid effect [33]. Moreover, puberty causes profound chemical and tissue composition changes, primarily because of gonadal sex steroids, which have an impact on glucose homeostasis and lipid metabolism, and which can increase insulin resistance, blood pressure, and cholesterol levels, making puberty a period of increased risk for developing a metabolic syndrome [35,36]. However, our study did not observe a tendency toward weight gain in the 12–17 age group. In contrast, a trend toward weight loss in the 12–17 age group during the induction treatment was documented. Another study evaluated the risk factors for weight loss during the induction phase of treatment in children with ALL or lymphoblastic lymphoma; in support of our findings, the results demonstrated that patients over 10 years old had a 4.3 times greater risk of weight loss than younger children [37].

The choice of ALL steroid backbone influences weight changes. A greater increase in weight observed in children receiving dexamethasone compared to prednisolone was previously reported [38]. All patients included in our study received dexamethasone during induction, and long-term administration of glucocorticoids is associated with rapid weight gain in children with ALL [39]. In our study, the youngest group of children appeared to be the most prone to weight gain; however, the increase in BMI did not reach statistical significance, most probably due to the small sample size.

Importantly, our research indicates that an increase in BMI during induction treatment predicts a higher BMI after completion of ALL maintenance therapy [40]. However, there are little data on the change in BMI during induction, which is one of the most challenging, albeit shortest, ALL treatment phases. Our results demonstrated that, even during a short four-week treatment period (as per protocol), a change in the body mass of the youngest and the oldest children can be expected. The findings further emphasise the importance of monitoring weight change during the induction period in all age groups, with the aim of taking early preventive measures for obesity or weight loss.

In our study, a statistically significant difference was detected in the average BMI *z*-scores between children presenting with temperatures of <38 °C or ≥38 °C at diagnosis in the 0–5 age group, suggesting that a higher BMI could be associated with an increased risk of fever at an ALL diagnosis. Obese patients have a larger amount of adipose tissue; therefore, they have elevated levels of adipocyte-produced hormones and cytokines, including those enhancing macrophage migration to the adipose tissue. Increases in adipose tissue volume and macrophage influx result in higher levels of fever-inducing proinflammatory cytokines (TNF-α, IL-1β, and IL-6) [41], already increased as a consequence of cancer. Fever in paediatric ALL can be caused by both leukaemia-related pyrogenic reactions, generating increased levels of cytokines from tumour cells or infiltrating mononuclear cells, and infection [7]. Only one patient had an infection confirmed by laboratory tests in our study, although other cases might have been not diagnosed. Otherwise healthy children with a higher BMI have been shown to be more likely to present with infections than children with a normal BMI, possibly because higher levels of leptin in obese children can disturb the immune system by affecting the quantity of lymphocytes and the function of phagocytes [42].

Our study results did not reveal any significant impact of nutritional status on treatment outcome, measured as an MRD status on day 29. However, other previously conducted studies, which cover more extended time periods, have reported different results, although findings on the association between obesity and leukaemia relapse and mortality have been inconsistent, as summarised in Table 3. Research suggests that poor outcomes might be caused by inadequate doses of chemotherapy in obese patients because they are dosed by body surface area [43]. Importantly, according to a few of these studies, an increased BMI is associated with a higher risk of relapse in patients over 10 years old, but not in younger children [43,44]. However, other studies did not discover any significant relationship between BMI *z*-scores and treatment-related complications or outcomes of ALL; therefore, further investigation is needed [45,46].

Our findings should be interpreted with caution because of the small sample size, which was the main limitation. Of note, most data were collected retrospectively; therefore, the investigation was limited to already existing information, and certain nutritional markers—e.g., vitamin D, glucose, and albumin—were not analysed as these tests were not performed for a significant proportion of the participants, although they can reveal information about a child’s nutritional status. Therefore, larger studies on the importance of malnutrition in the treatment of ALL are needed.

## 6. Conclusions

Our research emphasises the importance of careful nutritional status monitoring, as well as appropriate and timely corrections of nutritional support, with preschool and pubertal children as important target groups when considering interventions for weight gain and loss, respectively. Despite the study limitations, a relationship between BMI at diagnosis and fever at diagnosis in the 0–5 age group was identified, although no association was found between BMI at diagnosis and MRD (at the end of induction treatment) in any of the age groups.

## Figures and Tables

**Table 1:** Patient characteristics (*n* = 50).

	Total Patients No. (%)	Age Groups No. (%)			*p-*Value ^1^
		0–5 Years	6–11 Years	12–17 Years	
Number of Patients (%)	50 (100.0)	26 (52.0)	15 (30.0)	9 (18.0)	
Median age in years (range, IQR)	5.8 (0.4–15.8, 6.1)	4.1 (0.4–5.9, 2.3)	10.0 (6.7–11.6, 1.8)	15.1 (12.2–15.8, 2.8)	
Gender					
Male	28 (56.0)	16 (61.5)	8 (53.3)	4 (44.4)	0.652
Female	22 (44.0)	10 (38.5)	7 (46.7)	5 (55.6)	
Immunophenotype					
B-ALL	46 (92.0)	25 (96.2)	12 (80.0)	9 (100.0)	0.158
T-ALL	4 (8.0)	1 (3.8)	3 (20.0)	0 (0.0)	
Cytogenetics (B-ALL) ^2^					
Normal	2 (4.3)	0 (0.0)	2 (16.7)	0 (0.0)	0.367
Favourable risk	26 (56.5)	14 (56.0)	7 (58.3)	5 (55.6)	
High risk	2 (4.3)	1 (4.0)	0 (0.0)	1 (11.1)	
B-other	16 (34.8)	10 (40.0)	3 (25.0)	3 (33.3)	
Leukocyte count					
<50 × 10^9^/L	42 (84.0)	22 (84.6)	13 (86.7)	7 (77.8)	0.770
≥50 × 10^9^/L	8 (16.0)	4 (15.4)	2 (13.3)	2 (22.2)	
Induction type ^3^					
A	25 (51.0)	21 (84.0)	4 (26.7)	0 (0.0)	<0.001
B	24 (49.0)	4 (16.0)	11 (73.3)	9 (100.0)	
BMI *z*-scores at diagnosis					
Normal (−2 to 2)	47 (94.0)	24 (92.3)	14 (93.3)	9 (100.0)	0.509
Underweight (<−2)	1 (2.0)	0 (0.0)	1 (6.7)	0 (0.0)	
Overweight/obese (>2)	2 (4.0)	2 (7.7)	0 (0.0)	0 (0.0)	
BMI *z*-scores at end of induction					
Normal (−2 to 2)	40 (80.0)	18 (69.2)	14 (93.3)	8 (88.9)	0.172
Underweight (<−2)	4 (8.0)	2 (7.7)	1 (6.7)	1 (11.1)	
Overweight/obese (>2)	6 (12.0)	6 (23.1)	0 (0.0)	0 (0.0)	
Change in mean BMI *z*-score					
BMI *z*-scores at diagnosis (mean (SD))	0.2 (1.2)	0.4 (1.1)	−0.3 (1.3)	0.3 (1.0)	0.181
BMI *z*-scores at day 29 (mean (SD))	0.2 (1.5)	0.7 (1.7)	−0.3 (1.3)	−0.3 (1.0)	0.074
*p*-Value ^4^	0.996	0.273	0.856	0.005	
Fever at diagnosis					
<38 °C	31 (62.0)	15 (57.7)	11 (73.3)	5 (55.6)	0.554
≥38 °C	19 (38.0)	11 (42.3)	4 (26.7)	4 (44.4)	
Fever during induction treatment					
<38 °C	38 (76.0)	22 (84.6)	9 (60.0)	7 (77.8)	0.222
≥38 °C	12 (24.0)	4 (15.4)	6 (40.0)	2 (22.2)	
MRD on day 29 ^5^					
MRD negative	19 (38.8)	8 (30.8)	8 (53.3)	3 (37.5)	0.408
MRD positive	30 (61.2)	18 (69.2)	7 (46.7)	5 (62.5)	

^1^ Chi-square and Fisher’s exact tests were used to test the association between different categorical variables. One-way ANOVA was used to evaluate the difference in mean BMI *z*-scores among different age groups. ^2^ Favourable risk: high hyperdiploidy (≥51 chromosomes), ETV6–RUNX1; high risk: KMT2A rearrangement, low hypodiploidy (≤39 chromosomes), iAMP21. ^3^ Induction A and induction B were administered to NCI standard-risk patients and NCI high-risk patients, respectively. The patient aged <1 year was excluded. ^4^ A paired *t*-test was used to determine the change in mean BMI *z*-score from diagnosis to the end of induction treatment. ^5^ The patient who died was excluded. Abbreviations: ALL, acute lymphoblastic leukaemia; BMI, body mass index; IQR, interquartile range; MRD, minimal residual disease; SD, standard deviation.

**Table 2:** BMI *z*-scores at diagnosis and their relationship with fever and MRD.

	0–5 Years (*n* = 26)		6–11 Years (*n* = 15)		12–17 Years (*n* = 9)	
	BMI *z*-Scores at Diagnosis (Median (Range))	*p-*Value *	BMI *z*-Scores at Diagnosis (Median (Range))	*p*-Value *	BMI *z*-Scores at Diagnosis (Median (Range))	*p-*Value *
Fever at diagnosis						
<38 °C (*n* = 31)	−0.2 (−1.4, 1.3)	0.001	0.5 (−1.6, 0.9)	0.896	0.3 (−0.3, 0.8)	0.697
≥38 °C (*n* = 19)	1.5 (−0.7, 3.1)		0.0 (−3.6, 1.2)		0.7 (−1.2, 1.9)	
MRD at day 29						
Negative (*n* = 19)	0.2 (−0.7, 1.5)	0.680	−0.4 (−3.6, 1.2)	0.354	0.3 (−1.2, 1.9)	0.803
Positive (*n* = 30)	0.1 (−1.4, 3.1)		0.5 (−1.6, 0.9)		0.4 (−0.1, 1.6)	

* Independent-sample *t*-tests or two-sample Wilcoxon tests were used on the basis of the result of the Shapiro–Wilk normality test. Abbreviations: BMI, body mass index; MRD, minimal residual disease.

**Table 3:** Summary of main outcomes according to reported studies on BMI and ALL.

Authors	Number of Patients	Age Range	Diagnosis	Outcomes
Sun et al. [47]	82	1–10	ALL	Abnormally high BMI was associated with higher MRD measured on days 19 and 46.
Saenz et al. [44]	Cohort study: 181 Meta-analysis: 916	2–20	ALL, AML, CML	Overweight/obese patients ≥10 years showed a trend toward increased risk of relapse, which was not observed among children <10 years in the single-centre cohort study, although these results were not statistically significant. The meta-analysis revealed a statistically significant increase in mortality risk for overweight/obese patients.
Butturini et al. [43]	4260	2–20	ALL	The study revealed a higher chance of relapse in patients ≥10 years, who were obese at diagnosis, but no such trend in patients <10 years was noticed.
Hijiya et al. [45]	621	1.0–18.8	ALL	Event-free survival, overall survival, relapse incidence, and toxicities did not differ among different BMI categories.
Baillargeon et al. [46]	322	2–18	ALL	Obesity at diagnosis was not associated with decreased overall survival and event-free survival.

Abbreviations: ALL, acute lymphoblastic leukaemia; AML, acute myeloid leukaemia; CML, chronic myeloid leukaemia.

## Data Availability

Data sharing is not applicable to this article due to ethical restrictions.

## Abbreviations

ALL, acute lymphoblastic leukaemia; AML, acute myeloid leukaemia; ANOVA, analysis of variance; BMI, body mass index; CML, chronic myeloid leukaemia; IL, interleukin; IQR, interquartile range; MRD, minimal residual disease; NCI, National Cancer Institute; SD, standard deviation; TNF-α, tumour necrosis factor alpha; WHO, World Health Organisation.
